# DMFF-Net: a multi-scale feature fusion network based on DeepLabV3 for skin lesion segmentation

**DOI:** 10.3389/fmed.2025.1633209

**Published:** 2025-11-26

**Authors:** Tao Jiang, Shange Wang, Lin Xu, Ji Yin, Linshuai Zhang, Yujie Zhang, Jing Guo, Pengfei Zhang

**Affiliations:** 1 School of Intelligent Medicine, Chengdu University of Traditional Chinese Medicine, Chengdu, China; 2 School of Clinical Medicine, Chengdu University of Traditional Chinese Medicine, Chengdu, China

**Keywords:** skin lesion segmentation, DeepLabV3, multi-scale feature fusion, attention mechanism, medicine image segmentation

## Abstract

**Objective:**

In computer-aided medical diagnosis, precise skin lesion segmentation is crucial for the early detection and treatment of skin cancer. However, challenges such as unclear lesion boundaries, low contrast, and varying lesion shapes make accurate segmentation a difficult task. To address these challenges, we propose DMFF-Net, a multi-scale, multi-attention feature fusion network based on DeepLabV3, designed to improve the accuracy of skin lesion segmentation.

**Methods:**

DMFF-Net integrates several advanced modules to enhance segmentation performance. The network incorporates a Global Grid Coordinate Attention Module (GGCAM), which effectively fuses spatial and channel features to capture the complex relationships between local and global information. Additionally, a Multi-Scale Depthwise Separable Dilated Convolution (MDSDC) module is employed to strengthen multi-scale feature extraction, thereby preventing resolution degradation during convolution. A Mid-High Level Feature Fusion (MHLFF) module is also introduced to refine critical feature representations and suppress irrelevant information, thereby improving segmentation accuracy.

**Results:**

The proposed network was evaluated on four publicly available datasets: ISIC 2016, ISIC 2017, ISIC 2018, and *PH*^2^. The results show that DMFF-Net significantly outperforms existing advanced methods. Specifically, it achieves MIoU values of 89.31%, 91.47%, and 86.93% on the ISIC 2016, ISIC 2017, and ISIC 2018 datasets, respectively. Furthermore, the network achieves accuracy values of 95.62%, 97.33%, and 94.78%, and F1 scores of 96.93%, 94.91%, and 93.61%, respectively, demonstrating its robustness and effectiveness in skin lesion segmentation.

**Conclusion:**

The DMFF-Net model, with its multi-scale feature fusion and attention mechanisms, substantially improves skin lesion segmentation by preserving crucial spatial details and improving feature representation. Its superior performance on multiple datasets highlights its potential as a powerful tool for skin lesion diagnosis and provides an important reference for future advancements in medical image segmentation.

## 1 Introduction

Skin cancer is one of the most common malignancies worldwide. Malignant melanoma accounted for approximately 20% of the more than 1.5 million new instances of skin cancer that were reported globally in 2020 (1). Over recent decades, the incidence of malignant melanoma has steadily increased. However, despite the increasing rates of skin cancer, early diagnosis and treatment have shown relatively high success rates (2). Recent studies indicate that the five-year survival rate for early-stage melanoma can exceed 95% with timely treatment, further highlighting the crucial role of early intervention in skin cancer management. Skin cancer encompasses various types, and in its early stages, the morphological features are often subtle, making it difficult to accurately identify with the naked eye (3). As a result, dermatologists frequently rely on dermoscopy for early diagnosis. Notably, dermoscopy, a non-invasive imaging technique, has proven to improve diagnostic accuracy (4, 5). However, even experienced physicians may sometimes make subjective judgments or errors when interpreting dermoscopic images (6), and the manual diagnostic process can be time-consuming. To address these challenges, Computer-Aided Diagnostic (CAD) systems have been introduced for skin cancer diagnosis. Many of these systems rely on accurate segmentation of skin lesions to identify affected areas in images. Medical image segmentation has been revolutionized recently by Artificial Intelligence (AI) technologies, particularly machine learning and deep learning. These technologies exhibit greater accuracy and efficiency than conventional segmentation techniques. A notable example is the widespread application of CAD systems in medical image segmentation, which has significantly improved dermatologists' ability to analyze dermoscopic images (7).

Many machine learning methods were used to identify and categorize skin lesions; however, their feature extraction skills were restricted, and they frequently required manually created features. In contrast, deep learning techniques automatically extract and optimize features without manual intervention. By training on labeled data, these techniques autonomously learn high-level features, significantly improving the accuracy of skin lesion segmentation. For example, the U-Net design, which is based on an entire convolutional network, was presented by Ronneberger et al. (8) and has shown great promise in segmenting medical images. Subsequent research further developed the U-Net model, leading to variants such as U-Net++ (9) and UNet3+ (10). These improved models, especially U-Net++, effectively integrated features from different levels, constructing richer and more precise feature maps by carefully combining fine-scale differences in features. The DeepLabV3+ model, developed by Chen et al. (11), expanded upon the original DeepLabV3 by adding a straightforward and effective decoder module. This extension significantly improved segmentation accuracy, particularly in the fine details of object edges. Their model also thoroughly explored the Xception architecture and utilized depthwise separable convolutions to enhance the Atrous Spatial Pyramid Pooling (ASPP) and decoder modules, forming a balanced encoder-decoder network in terms of speed and performance. As well, Zhao et al. (12) introduced the Pyramid Scene Parsing Network (PSPNet), incorporating global contextual data via a pyramid pooling module, which allows for more efficient use of global context and improves the network's capacity to comprehend complex scenes. Chen et al. (13) proposed a network called TrUNet, combining the advantages of Transformers and Convolutional Neural Networks (CNN). By using Transformer and Res2Net as two branches of the encoder, TrUNet aims to extract rich global information to achieve precise segmentation of lesion areas in medical images. Sun et al. (14) presented LCAMix, an innovative approach for data augmentation in medical image segmentation. This approach mixes two images and their superpixel-based segmentation masks, integrating local and contour-aware strategies to improve segmentation accuracy. Besides the methods mentioned above, other studies have also focused on effective segmentation of skin lesions, as discussed in references (15–22), and have demonstrated promising results.

Although these techniques have yielded impressive outcomes in skin lesion segmentation, the task continues to be difficult because of issues like indistinct lesion boundaries, poor contrast, and irregular lesion shapes (23, 24). Moreover, existing CNNs have several significant limitations when addressing these issues. Firstly, fixed atrous rates lose fine boundary details; secondly, redundant feature channels are not adaptively weighted; lastly, pooling layers insufficiently model long-range dependencies, causing information loss.

In the study, a multiscale and multiattention feature fusion network, based on DeepLabV3 and referred to as DMFF-Net, is proposed to overcome obstacles in skin lesion segmentation operations. The method includes several key components: First, a Middle-High-Level Feature Fusion (MHLFF) module is introduced to enhance the network's capability of understanding complex scenes by integrating middle-level and high-level features. Second, the design of a Global Group Coordinate Attention Module (GGCAM) is employed in the decoder stage, following middle-high-level feature fusion, to generate attention maps utilizing global spatial information from the feature maps. That input feature maps are weighted using these attention maps, thereby improving feature representation. In addition, inspired by the ASPP module (25, 26), multi-scale feature fusion (27–30), and depthwise separable convolutions, the focus is on enhancing feature extraction while reducing computational complexity (31, 32). A Multi-Scale Depthwise Separable Dilated Convolution (MDSDC) module is incorporated in the encoder stage to extract features at various spatial scales. Unlike traditional convolutional networks that increase the receptive field by stacking multiple convolution layers, MDSDC significantly expands the receptive field by employing different dilation rates, while maintaining image resolution. This is critical for capturing a broader range of contextual information. Furthermore, global average pooling is combined to obtain global features from the entire image and integrate them with local features, helping the network better understand the relationships between different parts of the image and the whole. Most importantly, the output features from the multi-scale depthwise separable dilated convolution are concatenated along the channel dimension, with channel and spatial attention mechanisms applied to adjust the weights in both spatial and channel dimensions, enabling more accurate segmentation decisions. Four accessible skin disease datasets—ISIC 2016, ISIC 2017, ISIC 2018, and *PH*^2^—have been used in extensive experimental evaluations of the proposed DMFF-Net. Compared with State-Of-The-Art (SOTA) techniques, ablation trials have confirmed its efficacy. Here is a summary of the main contributions of the work:

1. Multi-scale feature integration for boundary preservation: By fusing low-level, mid-level, and high-level features, DMFF-Net effectively captures both global semantic context and fine lesion boundaries, particularly in challenging cases with blurry edges, low contrast, or irregular lesion shapes.
2. GGCAM: We introduce GGCAM in the decoder stage to refine feature representation by generating global attention maps. This design enhances critical lesion features while suppressing irrelevant background, leveraging both average and max pooling to capture comprehensive global context.
3. MDSDC: To balance performance and computational efficiency, MDSDC expands the receptive field using depthwise separable convolutions with varying dilation rates. This enables effective multi-scale feature extraction with significantly reduced parameters and FLOPs compared to traditional ASPP.
4. MHLFF: We design MHLFF to selectively combine mid-level local details with high-level semantic information. This complementary fusion mechanism improves robustness against background noise while retaining lesion details.

On four popular segmentation of skin tumor datasets, the experimental findings showed that our approach performed competitively when weighed against SOTA techniques and current mainstream methods.

The rest of the sections of this work are organized as follows: In addition to discussing current challenges and examining the use and advancements of attention mechanisms in medical image segmentation, Section 2 provides an overview of the state of research on skin lesion segmentation. The network design proposed in this study is described in detail in Section 3, emphasizing key techniques and novel features. The effectiveness of this study is demonstrated in Section 4, which presents experiments and comparative analyses with existing methods to validate the performance and efficiency of the proposed approach. Based on the findings in Section 4, Section 5 provides a more thorough analysis and summary of the proposed method, pointing out current limitations and outlining potential improvements. Finally, Section 6, the last part, concludes the work by highlighting the main contributions and findings of the research.

## 2 Relate work

### 2.1 Skin lesion segmentation

Accurate skin lesion segmentation is essential for patient diagnosis and care. The process requires a clear distinction between diseased regions and healthy skin. Traditionally, segmentation relied on various machine learning methods, including active contour models, thresholding methods (33–35), support vector machines (36), and edge detection techniques. However, these approaches were often limited by the complexity of image preprocessing and postprocessing, particularly in cases where the contrast between lesions and normal skin was low, which frequently resulted in imprecise segmentation boundaries.

To overcome these limitations, researchers introduced segmentation algorithms based on deep CNNs. These algorithms demonstrated superior performance with the segmentation of healthcare pictures without the need for extensive preprocessing, effectively improving the precision of segmenting skin lesions and providing more reliable clinical decision support for doctors. Long et al. (37) developed an image segmentation technique based on Fully Convolutional Networks (FCNs). The FCNs' design allowed this method to handle input images of any size, thereby enhancing its applicability and broadening its range of applications. Also, the optimization of the network structure reduced redundancy and increased operational efficiency. Nevertheless, this segmentation strategy might sacrifice some image details, indicating that there was room for improvement in terms of segmentation accuracy. Ronneberger et al. (8) introduced the U-Net model, which is built upon a FCN. This model had significant application value in the field of medical image segmentation. The U-Net model's structure comprised two parts: an encoder and a decoder. The encoder was responsible for extracting image features through downsampling to generate multiple feature maps, while the decoder reconstructed the spatial information of the image through upsampling to produce the final segmentation map. The main advantage of this model lies in its effective utilization of global positional information and contextual information, allowing U-Net to train efficiently even with a limited number of samples. The U-Net model has proven its worth in various medical image segmentation tasks, including the precise segmentation of neurons, cellular tumors, and HeLa cells. SkinNet (38) was a novel network architecture that improved upon the classic U-Net model. While maintaining the original model's strengths, it carefully designed the encoder structure. Specifically, SkinNet adopted the concept of expanded convolutions in the encoder part, which effectively increased the receptive field of each convolution kernel, enabling the network to capture a broader context. This design not only enhanced the network's ability to extract image features but also helped improve the model's recognition and segmentation performance of complex structures. Gu et al. (39) presented an innovative Context Encoder Network (CE-Net) that focuses on capturing detailed information and maintaining spatial feature integrity. The architecture comprises three primary components: a feature encoding module, a context extraction unit, and a feature decoding module. ASCU-Net, which was suggested by Tong et al. (40), segmented skin lesions using the idea of triple attention processes. These three attention processes worked together to help the network concentrate on more pertinent parts of the target.

In conclusion, the introduction of deep learning technology has brought revolutionary changes to the field of medical image segmentation. From the early techniques based on FCNs to the innovation of the U-Net model and the subsequent improvements with structures like SkinNet and ASCU-Net, these CNN-based algorithms have not only significantly improved segmentation accuracy but also simplified both the preprocessing and postprocessing complexities. In particular, the application of the U-Net model and its variants in skin lesion segmentation has provided strong technical support for accurate medical diagnosis and treatment. However, skin lesion segmentation still faces several challenges, including the irregular shape, varying sizes, low color contrast, and blurry boundaries of the lesion areas. These challenges have yet to be fully resolved. Therefore, this study proposes the multi-scale fusion network, DMFF-Net, aimed at effectively addressing these issues and providing more precise segmentation results, thus offering stronger technical support for the automated diagnosis and treatment of skin lesions.

### 2.2 Attention mechanism

Recently, attention mechanisms have advanced considerably in computer vision, with various techniques like channel attention, spatial attention, and self-attention (41, 42) gaining widespread use in image analysis and comprehension. Channel attention mechanisms allocate weights to each channel's feature map, emphasizing their relevance to basic information, allowing the network to pay more attention to feature maps rich in information. By calculating the feature correlations of every pixel in the spatial domain, spatial attention mechanisms concentrate on creating spatial-dimensional information and identifying significant details in a picture. The use of attention processes in the segmentation of medical images has greatly increased segmentation accuracy and efficiency. Particularly in learning context information, attention mechanisms can capture the intrinsic relationships between features and selectively enhance important information, thus optimizing the dependency relationships between spatial and channel dimensions. For instance, the Squeeze-and-Excitation (SE) module (42) is a widely used attention module that enhances the network's representational ability by explicitly modeling the dependencies between channels. The Bottleneck Attention Module (BAM) (43) is another simple yet effective attention module that can be used in conjunction with any feedforward convolutional neural network. It builds hierarchical attention at the bottleneck and infers attention maps via two distinct pathways (channel and spatial). Furthermore, the attention module created by Wei et al. (44), which automatically focuses on skin lesion features and suppresses irrelevant artifacts while extracting multi-scale discriminative features, is one example of a mixed-domain attention mechanism that combines the benefits of channel and spatial attention. FAT-Net was first presented by Wu et al. (45), who included an auxiliary transformer branch to capture global contextual information and substantial dependencies for skin lesion segmentation. TransUNet, a model that combines CNNs and transformers to efficiently collect local characteristics and global contextual information, respectively, was proposed by Chen et al. (46).

Previous studies have consistently demonstrated that incorporating attention mechanisms into CNNs can substantially enhance segmentation accuracy. Building on and synthesizing previous research, the GGCAM was developed. This module integrates multi-dimensional global information with attention weights, amplifying essential features to enhance segmentation performance. Furthermore, the MDSDC module was introduced, which extracts features across multiple scales and applies both channel and spatial attention mechanisms. By separately weighting these features, the MDSDC module effectively captures critical multi-scale feature information, contributing to improved segmentation accuracy.

## 3 Proposed method

### 3.1 DMFF-Net module framework

**Figure 1:**
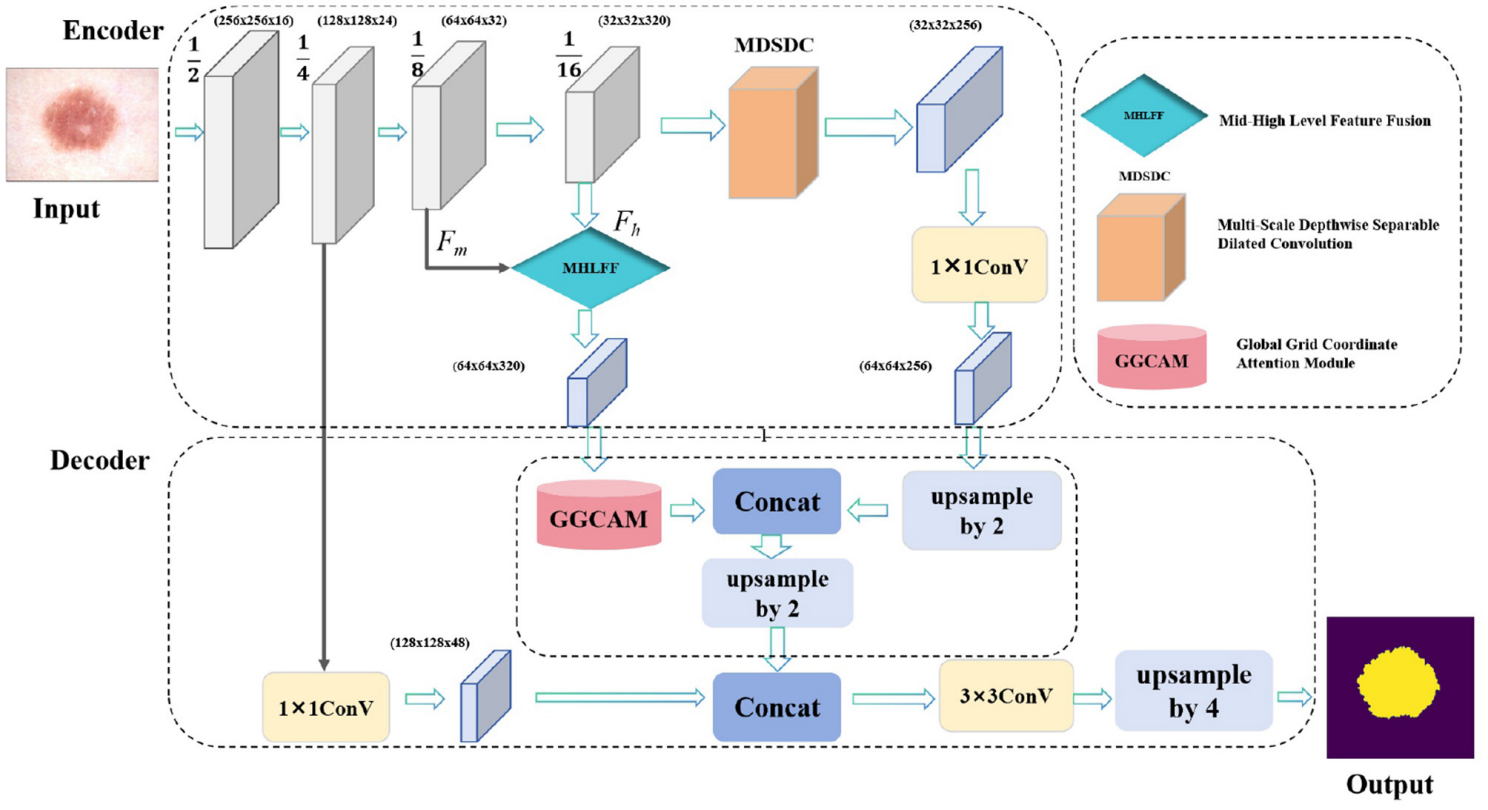
The proposed DMFF-Net architecture primarily consists of two main parts: the encoder and the decoder. Specifically, it includes three key modules: the MDSDC, the GGCAM, and the MHLFF.

The DMFF-Net model introduced in this study is aimed at addressing several key challenges in skin lesion segmentation, including fuzzy lesion borders, poor contrast, and the irregularity of lesion areas. As illustrated in Figure 1, our model has three main modules: the MDSDC module, the GGCAM module, and the MHLFF module. It is mainly composed of an encoder and a decoder. Firstly, the MHLFF module has been integrated into the original DeepLabV3 model. This module enhances the model's ability to understand and represent complex scenes by fusing mid-level and high-level features. Following the combination of mid-level and high-level features in the decoder stage, the GGCAM module was created and implemented. Using the global data from the feature maps' spatial dimensions (height and breadth), this module creates attention maps. To improve feature representation even more, these attention maps are then used to weight the input feature maps.

What's more, the MDSDC module was introduced in the encoder stage, which extracts features at different spatial scales. Unlike traditional methods that increase the receptive field by stacking multiple convolutional layers, the MDSDC module effectively expands the receptive field by using different dilated rates while maintaining the image resolution. This is crucial for capturing a broader context. Finally, the model uses channel attention and spatial attention mechanisms to weight channel and spatial features, respectively, capturing multi-scale key information and thereby improving the accuracy of skin lesion segmentation.

### 3.2 Multi-scale depthwise separable dilated convolution module

**Figure 2:**
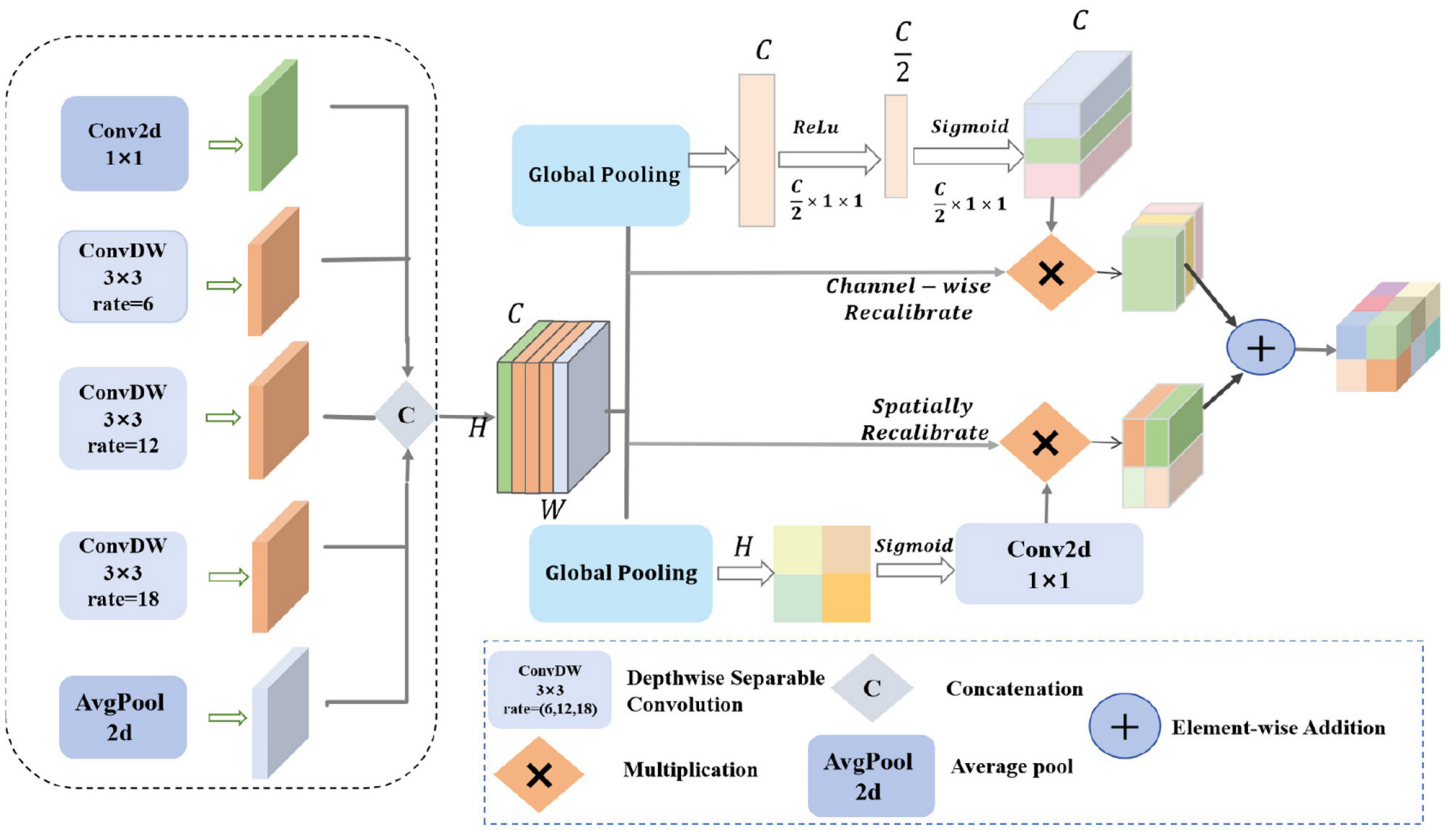
The structure diagram of the MDSDC module features diamonds with “C” to represent concatenation operations, diamonds with “ × ” to indicate multiplication operations, and diamonds with “+” to denote addition operations.

By integrating channel and spatial attention mechanisms and using depthwise separable convolutions with different dilation rates, the MDSDC module seeks to improve feature representation. For complicated visual tasks like semantic segmentation and object detection, this architecture is critical for gathering both extensive and detailed contextual information in images. Figure 2 provides an illustration of the architecture.

Firstly, our MDSDC architecture employs five parallel convolutional branches for multi-scale feature extraction. Each branch is configured with a different dilation rate to expand the receptive field and capture varying spatial information. The first branch uses a 1 × 1 convolution kernel to maintain the spatial scale and directly extract features. The second branch utilizes a 3 × 3 convolution kernel with a dilation rate of 6 to moderately expand the receptive field. The third branch employs a 3 × 3 convolution kernel with a dilation rate of 12 to further expand the receptive field and capture broader contextual information. The fourth branch uses a 3 × 3 convolution kernel with a dilation rate of 18 to provide the widest receptive field. The fifth branch applies global average pooling to extract global contextual features, enhancing the model's understanding of the overall layout. After these processes, the feature maps have the shape *H*×*W*×*C*. The Concat function is used to concatenate the different outputs along the channel dimension, resulting in a comprehensive feature map. Let the input feature map be **X**∈ℝ^*H*×*W*×*C*^, where *H, W*, and *C* are the height, width, and number of channels, respectively. The outputs of the five branches can be mathematically described as follows:


$$\left\{ \begin{array}{l} \;OP_{1}=f_{1\times 1Conv}(X)\\ OP_{2,3,4}=f_{3\times 3ConvDW}(X,r=[6,12,18])\\ \;OP_{5}=f_{upsample}\left(f_{1\times 1conv}\left(f_{avg-pool}(X)\right)\right) \end{array}\right.$$ (1)

$$\begin{array}{ll} F_{feature} & =\text{Concat}\left(\begin{array}{l} OP_{1},OP_{2},OP_{3},OP_{4},OP_{5} \end{array}\right) \end{array}$$ (2)

$$\begin{array}{ll} Output & =f_{1\times 1Conv}\left(F_{feature}\right) \end{array}$$ (3)

where *X* denotes the input feature map with dimensions *H*×*W*×*C*, and *OP*_*i*_ signifies the outcome of each distinct operation, *r* is the dilation ratio, and *f*_*aυg*−*pool*_ is the average pooling operation. The concatenation of various outputs in the channel dimension is known as the Concat function. Lastly, the final output feature map's shape remains *H*×*W*×*C*.

Two different kinds of attention mechanisms are then used to calibrate the combined feature map. To get the global features for each channel, the channel attention mechanism first applies global average pooling to the combined feature map. It then uses two fully linked layers with Sigmoid and ReLU activation functions, respectively, to learn the importance weights for each channel. Channel weighting is achieved by multiplying these weights by the original feature map on a per-channel basis.


$$\begin{array}{ll} F_{avg} & =\frac{1}{H\times W}\overset{H}{\underset{i=1}{\sum }}\overset{W}{\underset{j=1}{\sum }}F\left(i,j\right) \end{array}$$ (4)

$$\begin{array}{ll} w_{c} & =\sigma \left(\text{F}{\text{C}}_{2}\left(\text{ReLU}\left(\text{F}{\text{C}}_{1}\left(F_{avg}\right)\right)\right)\right) \end{array}$$ (5)

$$\begin{array}{ll} F_{c}^{\prime } & =w_{c}⊙F \end{array}$$ (6)

where *F*(*i, j*) represents the value of the feature map at position *i*, *j*, FC_1_ and FC_2_ are the two fully connected layers, σ denotes the Sigmoid activation function, and *w*_*c*_ is the importance weight for each channel. ⊙ represents element-wise multiplication, and $F_{c}^{\prime }$ is the feature map after channel weighting.

To create the spatial feature map, the spatial attention mechanism applies global pooling to the combined feature map along the channel dimension. It then uses a Sigmoid activation function and a 1 × 1 convolution to learn the importance weights for every spatial position. Spatial weighting is achieved by multiplying these weights element-wise by the original feature map.


$$\begin{array}{ll} F_{spatial} & =\left[\text{AvgPool}\left(F\right),\text{MaxPool}\left(F\right)\right] \end{array}$$ (7)

$$\begin{array}{ll} w_{s} & =\sigma \left({\text{Conv}}_{1\times 1}\left(F_{spatial}\right)\right) \end{array}$$ (8)

$$\begin{array}{ll} F_{s}^{\prime } & =w_{s}⊙F \end{array}$$ (9)

where [·, ·] denotes the concatenation operation, resulting in the spatial feature map *F*_*spatial*_, and $F_{s}^{\prime }$ is the feature map after spatial weighting.

Finally, the output feature maps from the channel attention and spatial attention mechanisms are fused through element-wise addition. The fused feature map is then combined with the original merged feature map through element-wise summation to integrate and enhance the relevant features. The enhanced feature map is subsequently processed through a 1 × 1 convolution layer for dimensionality reduction and integration, resulting in the final output feature map *F*_*output*_.


$$\begin{array}{ll} F_{fused} & =F_{c}^{\prime }+F_{s}^{\prime } \end{array}$$ (10)

$$\begin{array}{ll} F_{output} & =\text{Con}{\text{v}}_{1\times 1}\left(F_{fused}\right) \end{array}$$ (11)

where *F*_*fused*_ is the feature map obtained by fusing the channel and spatial features after weighting.

### 3.3 Global group coordinate attention module

**Figure 3:**
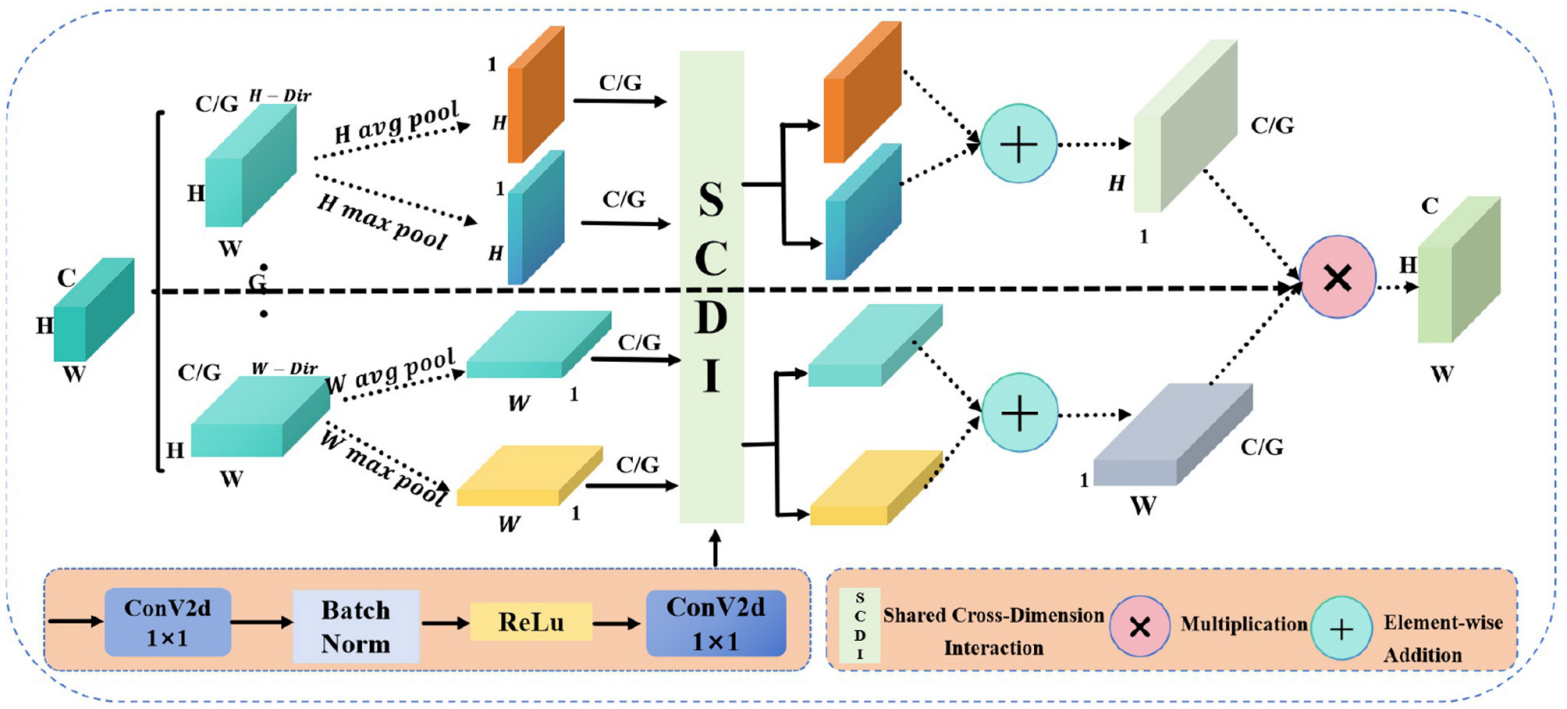
The structure diagram of the GGCAM shows circles with “+” to represent addition operations and circles with “ × ” to indicate multiplication operations.

Due to the traditional CNNs facing challenges in capturing global information across both spatial dimensions—height and width—when handling complex visual tasks, which limits their feature representation capabilities. Moreover, the importance of features varies across different channels and spatial locations, and conventional convolution operations are unable to dynamically adjust the significance of these features, making it difficult to highlight the most critical ones. Therefore, the proposed GGCAM aims to utilize the global information of feature maps in the spatial dimensions to generate attention maps. These attention maps are then used to weight the input feature maps, enhancing their representation capability. The specific structure of this module is illustrated in Figure 3. For the input feature map *F*, its dimensions are *B*×*C*×*H*×*W*. The feature map is divided into *G* groups based on the number of channels *C*, with each group containing *C*/*G* channels. The grouped feature maps are represented as:


$$\begin{array}{l} F_{g}=\left\{F_{1},F_{2},\ldots ,F_{G}\right\},\;F_{g}\in {\mathbb{R}}^{B\times \left(C/G\right)\times H\times W} \end{array}$$ (12)

where *F*(*i, j*) represents the value of the feature map at position (*i, j*), indicating the feature response or activation at that specific spatial location. And *B* is the batch size, *C* is the number of channels, and *H* and *W* are the height and width of the feature map, respectively.

For each grouped feature map *F*_*g*_, global average pooling and global max pooling are performed separately along the height and width directions, resulting in pooling outputs for both height and width.


$$\begin{array}{ll} F_{avg_{h}}^{g} & =\frac{1}{H}\overset{H}{\underset{i=1}{\sum }}F_{g}\left(i,j\right) \end{array}$$ (13)

$$\begin{array}{ll} F_{max_{h}}^{g} & =\overset{H}{\underset{i=1}{\max}}F_{g}\left(i,j\right) \end{array}$$ (14)

$$\begin{array}{ll} F_{avg_{w}}^{g} & =\frac{1}{W}\overset{W}{\underset{j=1}{\sum }}F_{g}\left(i,j\right) \end{array}$$ (15)

$$\begin{array}{ll} F_{max_{w}}^{g} & =\overset{W}{\underset{j=1}{\max}}F_{g}\left(i,j\right) \end{array}$$ (16)

where $F_{avg_{h}}^{g}$ and $F_{avg_{w}}^{g}$ represent the results of average pooling along the height and width dimensions, respectively, while $F_{max_{h}}^{g}$ and $F_{max_{w}}^{g}$ represent the results of max pooling along the height and width dimensions, respectively.

Additionally, a shared convolution layer is applied to process each grouped feature map. This convolution layer consists of two 1 × 1 convolution layers, batch normalization, and a ReLU activation function. First, we perform convolution operations on the pooling results:


$$\begin{array}{ll} F_{conv1}^{g} & =\text{ReLU}\left(\text{BN}\left(\text{Con}{\text{v}}_{1\times 1}\left(F_{avg_{h}}^{g}+F_{max_{h}}^{g}\right)\right)\right) \end{array}$$ (17)

$$\begin{array}{ll} F_{conv2}^{g} & =\text{ReLU}\left(\text{BN}\left(\text{Con}{\text{v}}_{1\times 1}\left(F_{avg_{w}}^{g}+F_{max_{w}}^{g}\right)\right)\right) \end{array}$$ (18)

where BN is the batch normalization operation. Then, the outputs of the convolution layers are summed, and the Sigmoid activation function is applied to generate the attention weights in the height and width directions:


$$\begin{array}{ll} A_{h}^{g} & =\sigma \left(F_{conv1}^{g}\right) \end{array}$$ (19)

$$\begin{array}{ll} A_{w}^{g} & =\sigma \left(F_{conv2}^{g}\right) \end{array}$$ (20)

where σ is the Sigmoid activation function.

In addition, the generated attention weights $A_{h}^{g}$ and $A_{w}^{g}$ are applied to the input grouped feature map *F*_*g*_, performing weighting in the height and width directions, respectively, to obtain the weighted feature maps $F_{h\text{\_}w}^{g}$ and $F_{w\text{\_}w}^{g}$. Finally, the attention weights are expanded in the height and width directions to match the dimensions of the input feature map, resulting in the final output feature map. In this way, by grouping and applying global information weighting across the spatial dimensions, the enhanced feature map *F*_*output*_ is ultimately generated.


$$\begin{array}{ll} F_{h\text{\_}w}^{g} & =A_{h}^{g}⊙F_{g} \end{array}$$ (21)

$$\begin{array}{ll} F_{w\text{\_}w}^{g} & =A_{w}^{g}⊙F_{g} \end{array}$$ (22)

$$\begin{array}{ll} F_{output}^{g} & =F_{h\text{\_}w}^{g}+F_{w\text{\_}w}^{g} \end{array}$$ (23)

where $F_{h\text{\_}w}^{g}$ and $F_{w\text{\_}w}^{g}$ represent the weighted feature maps in the height and width directions, respectively, and *F*_*output*_ is the enhanced output feature map.

### 3.4 Middle-high-level feature fusion module

**Figure 4:**
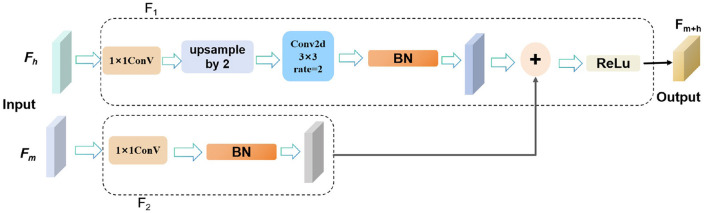
The structure diagram of the MHLFF illustrates the integration of features from different levels.

To capture rich semantic and contextual information, facilitate feature sharing across different layers of the network, and enhance the network's learning ability and generalization capabilities, we fuse the image features from the seventh layer and the final layer of the backbone network. As shown in Figure 4. Specifically, the mid-level features *F*_*m*_ from the seventh layer are input into the *F*_1_ module, which processes them using a 1 × 1 convolution followed by batch normalization to produce output *OP*_1_. Simultaneously, the high-level features *F*_*h*_ from the final layer are processed in the *F*_2_ module, where a 1 × 1 convolution is applied for dimensionality reduction, followed by upsampling and a 3 × 3 dilated convolution with a dilation rate of 2, concluded by batch normalization to yield output *OP*_2_.

The outputs *OP*_1_ and *OP*_2_ are then combined through element-wise addition to effectively merge the strengths of both mid-level and high-level features. Finally, the fused output undergoes ReLU activation to produce the final output *F*_*m*+*h*_. This integrated process enhances the network's ability to capture complex semantic and contextual details, improving performance across various visual tasks.


$$\begin{array}{ll} {\text{O}\text{P}}_{1} & =\text{BatchNorm}\left(\text{Con}{\text{v}}_{1\times 1}\left({\text{F}}_{m}\right)\right) \end{array}$$ (24)

$$\begin{array}{l} {\text{O}\text{P}}_{2}=\text{BatchNorm}\\ \;(\text{Con}{\text{v}}_{3\times 3,\text{dilation}=2}\left(\text{Upsample}\left(\text{Con}{\text{v}}_{1\times 1}\left({\text{F}}_{h}\right)\right)\right)) \end{array}$$ (25)

$$\begin{array}{ll} {\text{F}}_{\text{fused}} & ={\text{O}\text{P}}_{1}+{\text{O}\text{P}}_{2} \end{array}$$ (26)

$$\begin{array}{ll} {\text{F}}_{m+h} & =\text{ReLU}\left({\text{F}}_{\text{fused}}\right) \end{array}$$ (27)

where *F*_*m*_ represents the image features from the seventh layer, also known as mid-level features. and *F*_*h*_ represents the image features from the final layer, also known as high-level features.

## 4 Experience

### 4.1 Dataset

**Figure 5:**
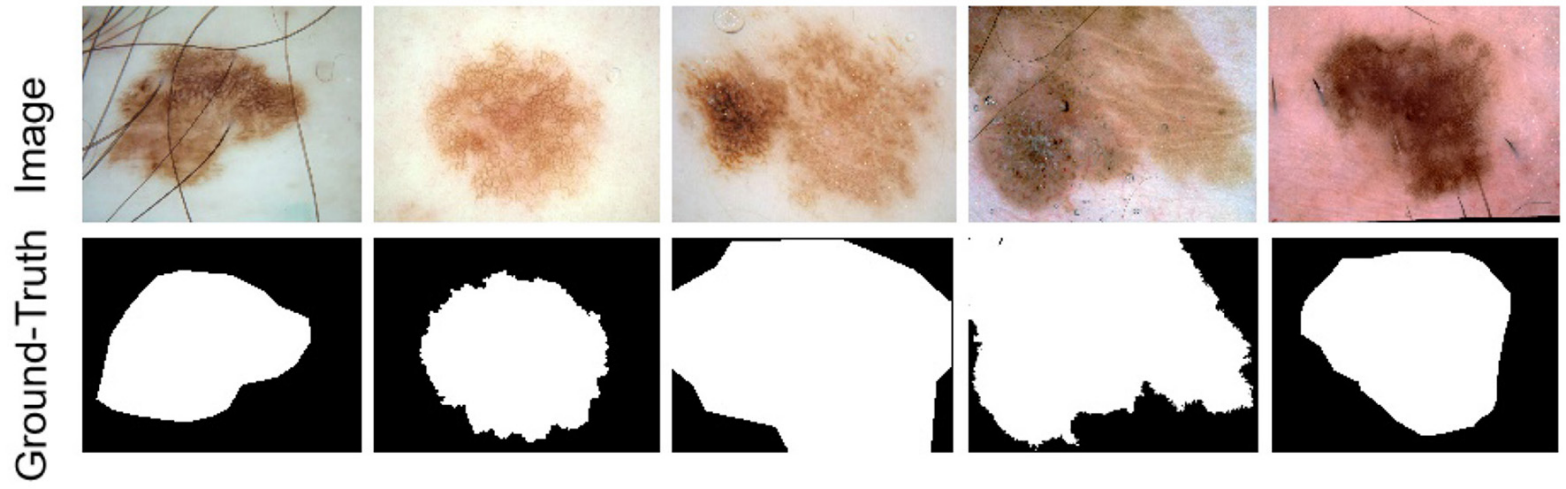
Example images of skin lesions and their corresponding label images from the ISIC dataset.

Four publicly accessible datasets from the International Skin Imaging Collaboration (ISIC) were used in this study to assess our methodology: ISIC 2016 (47), ISIC 2017 (48), ISIC 2018 (49), and the *PH*^2^ dataset (50). While Figure 5 displays some photos and the label maps that correlate to them. These tests assessed how well the suggested approach performed in tasks involving the segmentation of skin lesions.

1. ISIC 2016 dataset: includes 379 test images in JPEG format and 900 training images.
2. ISIC 2017 dataset: consists of 600 test photos, 150 validation photos, and 2,000 training photos.
3. ISIC 2018 dataset: The 2,596 RGB images in this dataset, which is intended for skin lesion segmentation, are randomly divided into 70% for training, 10% for validation, and 20% for testing.
4. *PH*^2^ dataset: Includes 200 skin lesion images and is frequently used to assess the generalization ability of models in lesion diagnosis tasks.

### 4.2 Experience details

All experiments were conducted on a workstation equipped with the PyTorch deep learning framework and an NVIDIA GeForce RTX 3080 GPU. The model was trained for 180 epochs with a batch size of 8 and an input image resolution of 512 × 512. We used stochastic gradient descent (SGD) with a momentum of 0.9 and an initial learning rate of 7 × 10^−3^, which was decayed using a polynomial learning rate schedule to ensure stable convergence. To improve generalization, we applied several data augmentation strategies, including random horizontal and vertical flipping as well as random cropping. These settings ensured both stable optimization and increased robustness against overfitting.

### 4.3 Evaluation metrics and loss function

Mean Intersection over Union (MIoU), pixel-wise Accuracy (Acc), F1 score, Mean Recall (MRecall), and Precision are the five primary assessment metrics used in this work, which are in line with the methodology of the majority of medical picture segmentation techniques. The following are the formulas used to calculate these metrics:


$$\begin{array}{ll} \text{Acc} & =\frac{TP+TN}{TP+TN+FP+FN} \end{array}$$ (28)

$$\begin{array}{ll} \text{MIoU} & =\frac{1}{N}\overset{N}{\underset{i=1}{\sum }}\frac{TP_{i}}{TP_{i}+FP_{i}+FN_{i}} \end{array}$$ (29)

$$\begin{array}{ll} \text{Recall} & =\frac{TP}{TP+FN} \end{array}$$ (30)

$$\begin{array}{ll} \text{Precision} & =\frac{TP}{TP+FP} \end{array}$$ (31)

$$\begin{array}{ll} F1 & =2\cdot \frac{\text{Precision}\cdot \text{Recall}}{\text{Precision}+\text{Recall}} \end{array}$$ (32)

Where TP, TN, FP, and FN represent true positives, true negatives, false positives, and false negatives, respectively. The values of all evaluation metrics range from 0 to 1, with values closer to 1 indicating better segmentation performance and values further from 1 indicating poorer performance.

In addition, the loss function for the binary classification segmentation module in this work was Binary Cross-Entropy (BCE) loss (51). As shown in Equation 33, this loss function is especially appropriate for binary classification problems since it quantifies the difference between the true labels and the anticipated class. The goal of the training process was to reduce this loss function in order to improve the binary classification accuracy of the model. Furthermore, the model parameters were updated using the Adam optimization algorithm, which iteratively optimized the loss value for better segmentation performance. The following is the definition of the BCE loss:


$$\begin{array}{ll} L & =-\frac{1}{N}\overset{N}{\underset{i=1}{\sum }}\left[y_{i}\log\left(p_{i}\right)+\left(1-y_{i}\right)\log\left(1-p_{i}\right)\right] \end{array}$$ (33)

where L is the Binary Cross-Entropy loss value, *N* is the total number of samples, *y*_*i*_ is the true label of the *i*^*th*^ sample (0 or 1), and *p*_*i*_ is the probability that the model predicts the *i*^*th*^ sample as the positive class.

### 4.4 Comparing with SOTA methods

We compare the proposed method with other recent CNN-based skin lesion segmentation techniques to show its effectiveness. Specifically, we replicated, trained, and comprehensively evaluated seven models on three different datasets. To ensure the reliability and representativeness of the results, the performance of the models was evaluated by quantitative metrics and visual inspection. Accurate quantitative metrics were used to assess segmentation performance, while visualization illustrated the effectiveness of each model in processing skin lesion images. The following table summarizes the quantitative results for each dataset, with the best values highlighted in bold and the next best values underlined. In addition, the visualization comparison graph depicts the segmentation results of each method along with ground truth annotations. Moreover, in the visualized result plots, the problem areas of each network model in the segmentation result plots are marked with red circles or boxes. This multidimensional analysis allows for a visual assessment of the strengths and weaknesses of each model and highlights the competitive advantages of the proposed models on different datasets.

#### 4.4.1 Performance on ISIC 2016 dataset

Table 1 reports the segmentation performance of DMFF-Net and seven SOTA methods on the ISIC 2016 dataset. Overall, DMFF-Net achieves the best performance across almost all metrics, with an mIoU of 89.31%, accuracy of 96.93%, and F1 score of 95.62%. Compared with DCSAU-Net, the strongest competing baseline, DMFF-Net improves mIoU and accuracy by 4.19% and 1.01%, respectively. Notably, DMFF-Net attains the highest precision (95.03%) and demonstrates a strong balance between recall (93.53%) and F1, which indicates its ability to accurately capture lesion boundaries while reducing false positives. These results confirm that DMFF-Net not only surpasses classical architectures such as U-Net and U-Net++, but also maintains clear advantages over more recent SOTA approaches (e.g., EGEUNET, MALUNET, and DCSAU-Net), particularly in terms of segmentation accuracy and robustness.

**Table 1:** Skin lesion segmentation performances of different networks on ISIC 2016.

**No**.	**Method**	**MIoU (%)**	**Acc (%)**	**F1 (%)**	**MRecall (%)**	**Precision (%)**
1	Unet	84.98	96.68	91.51	93.13	91.33
2	Unet++	84.64	96.44	91.13	92.41	91.92
3	DeeplabV3+	85.09	96.42	91.42	**94.11**	90.49
4	FPN	85.29	96.77	91.56	93.41	91.43
5	EGEUNET	84.01	95.14	91.31	89.08	93.66
6	MALUNET	82.61	94.81	90.48	86.01	**95.43**
7	DCSAU-Net	85.12	95.92	91.59	90.69	94.12
8	DMFF-Net(Ours)	**89.31**	**96.93**	**95.62**	93.53	95.03

The bold values indicate the best performance among the compared methods. The underlined values denote the second-best performance among the compared methods.

**Figure 6:**
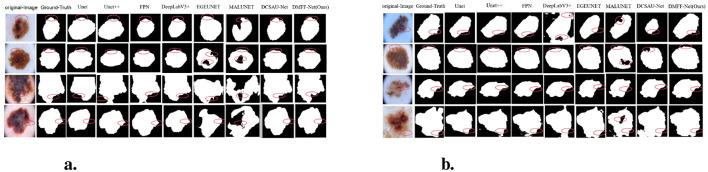
Visual comparison with different methods on ISIC 2016 **(a)** and 2017 **(b)** datasets.

Furthermore, Figure 6a shows the segmentation results on the ISIC 2016 test dataset. In comparison to MALUNET and FPN, the segmentation outputs of DMFF-Net were shown to be closer to the genuine labels. Interestingly, DMFF-Net outperformed DCSAU-Net in addressing unclear lesion regions and irregular boundaries. It is possible to conclude that DMFF-Net performed better in the skin lesion segmentation job on the ISIC 2016 test dataset by combining the quantitative results in Table 1 with the visual comparisons in Figure 6a.

#### 4.4.2 Performance on ISIC 2017 dataset

Table 2 summarizes the segmentation results of DMFF-Net and seven baseline models on the ISIC 2017 dataset. DMFF-Net consistently achieves the best performance across all five evaluation metrics. In particular, DMFF-Net reaches an mIoU of 91.47%, which is 6.35% higher than DCSAU-Net, the strongest competing baseline. It also records the highest accuracy (97.33%), F1 score (94.91%), mean recall (95.17%), and precision (95.76%). Compared with classical networks (e.g., U-Net and U-Net++), DMFF-Net yields an improvement of over 8% in mIoU, demonstrating its superior ability to capture fine-grained lesion boundaries. Moreover, the balanced gains in both recall and precision indicate that DMFF-Net not only reduces false negatives but also minimizes false positives, ensuring reliable segmentation across diverse lesion appearances.

**Table 2:** Skin lesion segmentation performances of different networks on ISIC 2017.

**No**.	**Method**	**MIoU (%)**	**Acc (%)**	**F1 (%)**	**MRecall (%)**	**Precision (%)**
1	Unet	80.01	96.64	87.43	86.53	90.23
2	Unet++	82.94	96.90	88.79	88.88	90.46
3	DeeplabV3+	78.58	96.13	86.89	90.62	85.23
4	FPN	81.52	96.61	87.21	89.07	87.45
5	EGEUNET	78.07	96.10	87.68	83.02	92.90
6	MALUNET	76.80	95.58	86.88	87.41	86.35
7	DCSAU-Net	88.32	95.33	91.49	91.69	95.12
8	DMFF-Net(Ours)	**91.47**	**97.33**	**94.91**	**95.17**	**95.76**

The bold values indicate the best performance among the compared methods.

Moreover, Figure 6b shows a visual comparison of a number of common atypical skin lesions. Unlike other models, DMFF-Net integrates mid-level and high-level feature fusion with a mechanism that generates attention maps using global information in the spatial dimension. This mechanism effectively assigns weights and enhances feature representation. What's more, DMFF-Net aggregates both global and local information while controlling the receptive field for feature extraction. These characteristics enable the model to handle finer details, particularly in segmenting blurred edges, allowing for cleaner and more distinct extraction of edge features. Notably, DMFF-Net outperformed other SOTA models in segmenting lesion areas. Overall, the segmentation results of DMFF-Net are more aligned with the original annotated images, demonstrating superior expressiveness and robustness.

#### 4.4.3 Performance on ISIC 2018 dataset

The ISIC 2018 dataset is larger and more diverse than ISIC 2016 and 2017, including a broader spectrum of lesion categories and presenting greater segmentation challenges. After retraining on this dataset, the results in Table 3 demonstrate that DMFF-Net delivers the most competitive performance across all evaluation metrics. Specifically, DMFF-Net achieves an mIoU of 86.93%, surpassing the strong baseline DCSAU-Net by 5.37%. It also records the best F1 score (93.61%), mean recall (92.49%), and precision (93.30%), confirming its superior ability to balance sensitivity and specificity. Although the overall accuracy is slightly lower than U-Net++ and DeepLabV3+, DMFF-Net's consistently higher mIoU and F1 show that it captures lesion boundaries more accurately, a critical factor in medical image segmentation.

**Table 3:** Skin lesion segmentation performances of different networks on ISIC 2018.

**No**.	**Method**	**MIoU (%)**	**Acc (%)**	**F1 (%)**	**MRecall (%)**	**Precision (%)**
1	Unet	74.36	94.15	82.98	90.25	79.50
2	Unet++	79.07	95.21	85.07	87.95	85.11
3	DeeplabV3+	77.86	95.06	85.48	92.09	81.61
4	FPN	76.21	94.73	84.57	90.44	82.74
5	EGEUNET	78.34	94.18	87.85	86.46	89.29
6	MALUNET	76.10	93.54	86.43	84.47	88.47
7	DCSAU-Net	81.56	94.84	88.54	89.25	91.39
8	DMFF-Net(Ours)	**86.93**	**94.78**	**93.61**	**92.49**	**93.30**

The bold values indicate the best performance among the compared methods.

**Figure 7:**
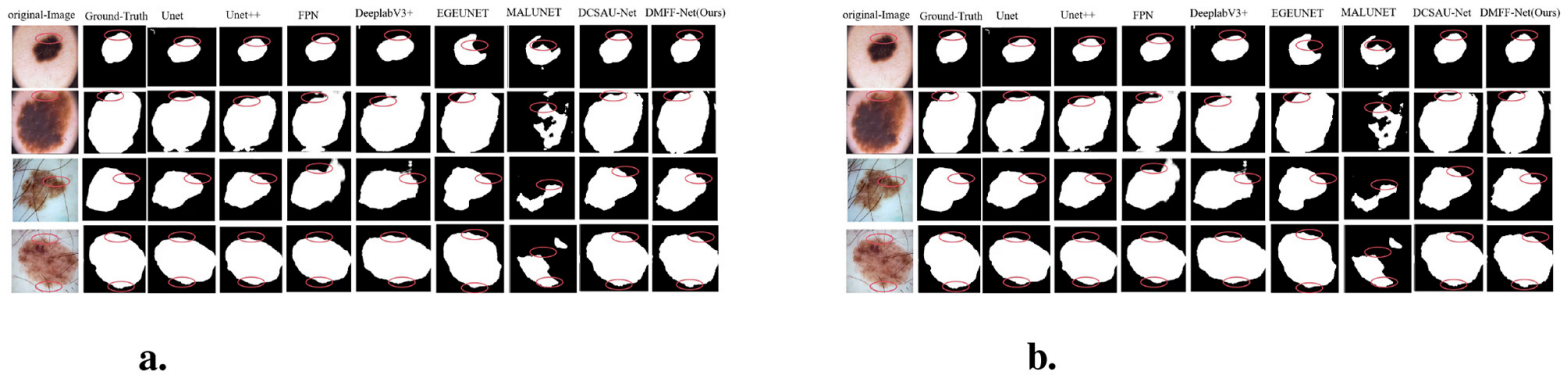
**(a)** Visual comparison with different methods on the ISIC 2018 dataset. **(b)** Cross-validation visualization comparison on ISIC 2017 and *PH*^2^ datasets.

Figure 7a presents graphic examples to give a more intuitive demonstration of the segmentation findings across several models on the ISIC 2018 dataset. Images with uneven lesion regions and hazy boundaries are displayed in the first and second rows. As illustrated, the proposed method demonstrates superior performance in handling irregular and blurred boundaries, largely due to the introduction of the mid-level and high-level feature fusion module, which enables precise extraction of edge detail features. The third and fourth rows present cases involving hair occlusion. With the use of the MDSDC module and GGCAM module, the model can perform weighted extraction of both channel and spatial features, effectively fusing feature information along the height and width dimensions. As shown in the images, our method also outperforms others in addressing hair occlusion, further validating the robustness and accuracy of the model.

Overall, thanks to the introduction of these innovative modules, our method significantly outperforms other SOTA models on the ISIC 2018 dataset, particularly in handling complex, blurred boundaries.

### 4.5 Cross-dataset assessment

The ISIC 2017 and *PH*^2^ datasets were used as testing platforms for cross-dataset validation in order to assess the DMFF-Net model's generalization ability. To find the ideal parameters, the model was initially trained and verified using the ISIC 2017 dataset. The model was then tested on the *PH*^2^ dataset to evaluate its generalization performance.

For the purpose of ensuring a comprehensive evaluation, the performance of other models, including FAT-Net (45), EIU-Net (52), DCSAUNet (53), EGEUNet (54), and MALUNet (55), was compared. FAT-Net integrates a transformer branch crafted to efficiently capture extensive dependencies and global context. The model utilizes a memory-optimized decoder along with a feature adaptation module that strengthens feature fusion across adjacent layers by emphasizing relevant channels and minimizing background noise, ultimately enhancing segmentation outcomes. On the other hand, throughout its different stages, EIU-Net leverages inverse residual blocks and Efficient Pyramid Squeeze Attention (EPSA) blocks as essential components within its encoder. These blocks are designed to enhance feature extraction by selectively focusing on relevant spatial and channel information, enabling the model to more effectively capture complex details in medical imaging tasks. By integrating these advanced modules, EIU-Net aims to improve the overall accuracy and robustness of the segmentation process. ASPP and soft pooling techniques are applied for downsampling after the final encoder, while feature maps from different decoders are integrated to obtain multi-scale information, further enhancing skin lesion segmentation accuracy.

In contrast, our DMFF-Net model builds upon these models by incorporating the extraction of channel and spatial feature information, along with fusion and importance weighting in both height and width directions, achieving comprehensive information extraction. Comparative analysis of the predicted segmentation images post-training (as shown in Figure 7b) reveals that our DMFF-Net model retains details and handles edges more closely to the original annotated images. These findings confirm the model's effectiveness in addressing the complexities of skin lesion segmentation, demonstrating its strength in accurately capturing subtle lesion features.

**Figure 8:**
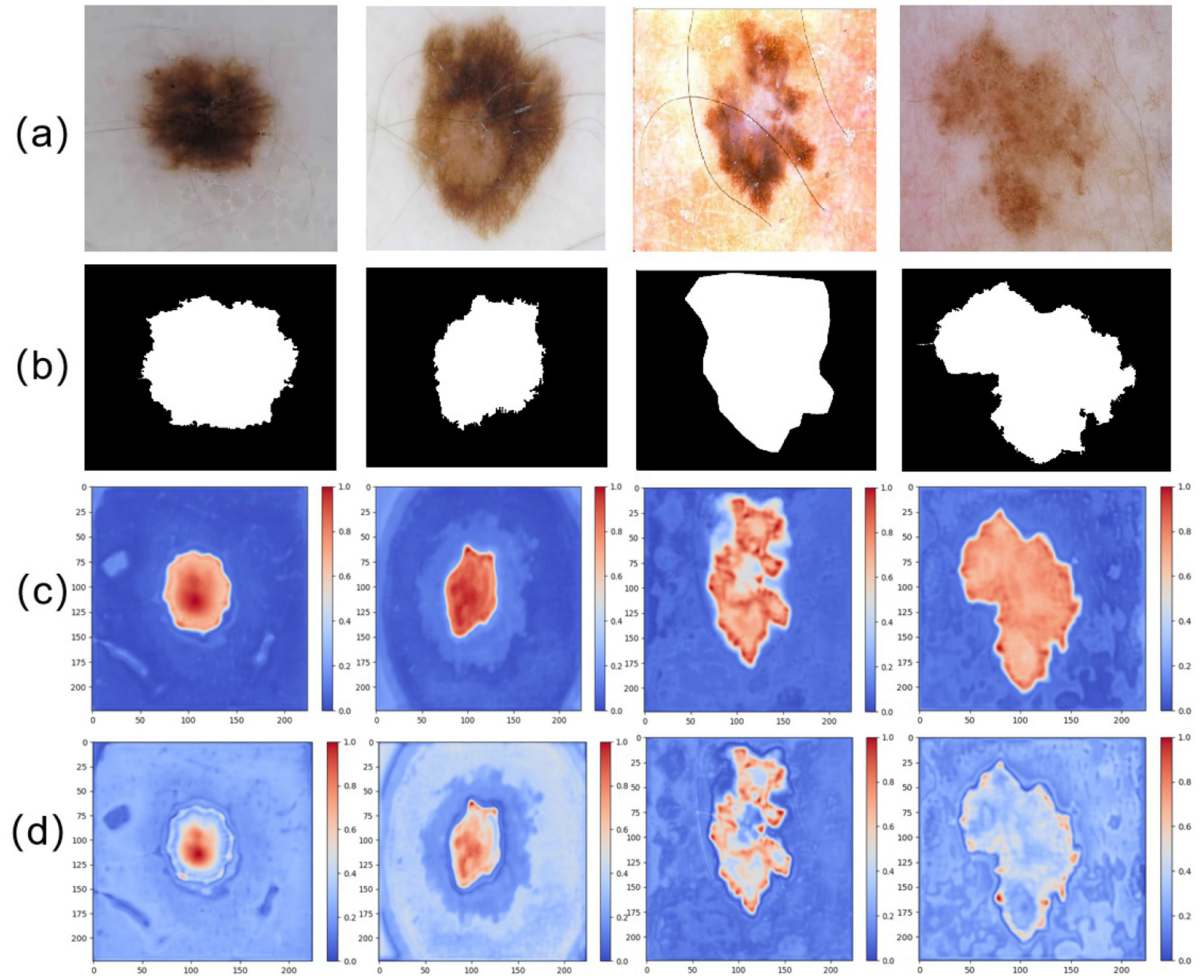
Comparison of different attention maps extracted at various stages: **(a)** input image, **(b)** GT image, **(c)** attention heatmap from the final layer of the decoder, and **(d)** attention heatmap from the final layer of the encoder.

Besides, to enhance the interpretability of the network and visualize the regions of interest at different stages, particularly within the encoder and decoder, we performed attention heatmap visualizations on the features captured by the final layers of both the encoder and decoder. The results are shown in Figure 8. As illustrated in (d), the attention map from the encoder highlights its focus on the boundary regions of the skin lesions. This suggests that the encoder plays a crucial role in improving the network's ability to capture the edges of the lesions, thereby enabling more accurate delineation of lesion boundaries. Building upon this, (c) demonstrates the attention map of the final layer of the decoder. By integrating the fine-grained feature capturing of the lesion boundaries in the encoder with the feature extraction of the internal lesion regions in the decoder, the model ultimately achieves a complete segmentation of the lesion area.

### 4.6 Ablation experiment

In experimental design, the comparison of individual components within an algorithm is crucial for accurately evaluating their contributions to overall performance (56). As such, a series of incremental ablation experiments was performed to evaluate the impact of each component within the proposed network, using the ISIC 2017 dataset to systematically analyze its contributions to overall performance. DeeplabV3 was initially used as the baseline model, with different modules—such as GGCAM, MDSDC, and MHLFF—progressively introduced as the primary feature extraction components. By progressively adding these modules, their influence on the network's general effectiveness was observed through analysis of the resulting performance variations. This approach provided valuable insights into the individual contributions of each module in optimizing the network and enhancing segmentation accuracy.

**Figure 9:**
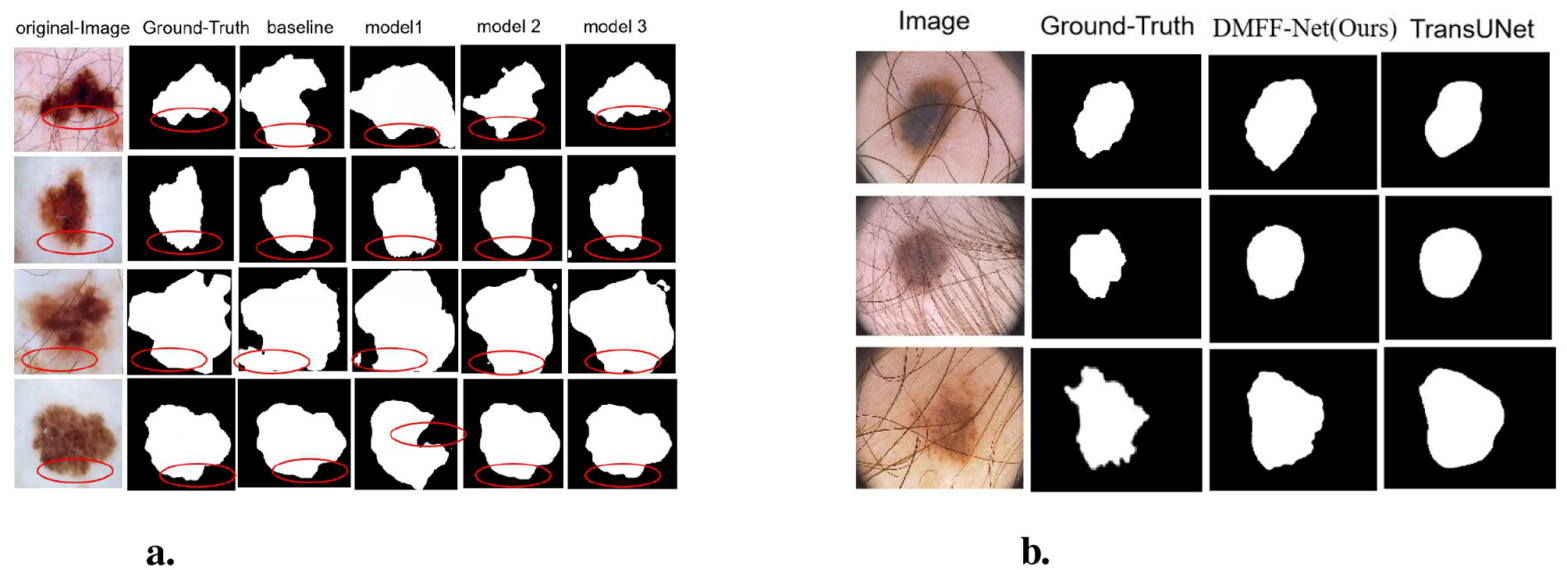
**(a)** Visualization comparison of ablation experiments on the ISIC 2017 dataset. **(b)** Qualitative comparison between DMFF-Net and TransUNet on PH2 images with severe artifacts.

Table 4 and Figure 9a illustrate the segmentation performance and visual results of different models. The baseline model represents the segmentation performance of the original DeepLabV3, while model 1 indicates the network performance and segmentation results after adding the MHLFF module to the baseline. Model 2 shows the performance after adding the MDSDC module to model 1, and model 3, which incorporates the GGCAM module added to model 2, represents the final model proposed in this study. As shown in Table 4 and Figure 9a, in the first row of experiments, the original image contains slight hair occlusion with blurred and irregular lesion edges. Although the evaluation metrics of the baseline model are higher than those of model 1, the segmentation results of both models still show a significant gap compared to the Ground Truth (GT). This may be due to their limited performance in handling hair occlusion and irregular lesions. However, with the addition of the MDSDC and GGCAM modules—in models 2 and 3—the evaluation metrics improve progressively, and the segmentation results increasingly resemble the GT. This indicates that the MDSDC and GGCAM modules have stronger abilities to extract complex edge information and process blurred areas. Similarly, in the third row of experiments in Figure 9a, although the original image contains slight hair occlusion and has highly blurred lesion edges, the lesion shape is relatively regular. As a result, the segmentation results from all models are generally similar to the GT. However, model 3 performs better in extracting fine details along the blurred edges, providing a more accurate restoration of the details.

**Table 4:** Comparison of ablation experiments for the main module in the DMFF-Net, where Params is the number of parameters and FLOPs is Floating Point Operations. Mark the best result in red font and the Baseline is DeepLabV3.

**Model**	**MIoU (%)**	**Acc (%)**	**F1 (%)**	**Params (M)**	**FLOPs (G)**
Baseline	89.34	96.08	95.31	6.870	38.59
Model1	89.19	96.05	94.90	4.522	29.47
Model2	90.9	97.18	95.1	6.470	31.48
Model3	91.47	97.33	94.91	4.520	29.47

The underlined values denote the second-best performance among the compared methods. Red-colored values indicate the best performance.

In the second and fourth rows of Table 4, where the original image edges are also blurred, the performance of the models progressively improves from the baseline to model 3, especially in the extraction of edge details, showing significant advantages. It is worth noting that Table 4 shows a reduction in both parameter count and computational complexity for models 1, 2, and 3 compared to the baseline. The lowest parameter count is 4.52M, and the FLOPs is 29.47G. However, despite the reduction in parameter count, the overall computational complexity remains relatively high. Thus, future research could focus on reducing computational complexity and further lightening the model, making it more suitable for resource-constrained environments.

To further verify the independent contribution of each module, we conducted single-module ablation experiments under the same setting (Table 5). Replacing ASPP with MDSDC slightly reduces MIoU, ACC, and F-score (89.19%, 96.05%, and 94.9%) compared with ASPP (90.96%, 97.2%, and 95.12%), but the number of parameters and FLOPs drops markedly to 4.522, M and 29.479, G, demonstrating that MDSDC can effectively decrease computational cost while maintaining comparable accuracy. When MHLFF and GGCAM are individually added to the baseline, MHLFF yields similar accuracy but significantly fewer parameters and FLOPs (4.522, M , 29.47, G), whereas GGCAM achieves more noticeable accuracy gains (MIoU 91.34%, ACC 97.68%, and F-score 97.1%) with reduced computational overhead. Combining the two modules delivers superior performance in both accuracy (MIoU 91.05%, ACC 97.21%) and efficiency (4.520, M, 29.47, G), verifying the complementarity of GGCAM and MHLFF: the former enhances global semantic context, while the latter strengthens multi-scale feature fusion, jointly improving segmentation performance and reducing computational cost.

**Table 5:** Single-module ablation study on ISIC dataset.

**Model**	**MIoU(%)**	**ACC(%)**	**F-score(%)**	**#Params(M)**	**FLOPs(G)**
Baseline	89.34	96.08	95.31	6.870	38.59
Baseline+ASPP	90.96	97.20	95.12	6.870	38.593
Baseline+MDSDC	89.19	96.05	94.90	4.522	29.479
Baseline+MHLFF	89.19	96.05	94.91	4.522	29.47
Baseline+GGCAM	91.34	97.68	97.10	6.476	31.481
Baseline+GGCAM+MHLFF	91.05	97.21	95.10	4.520	29.47

In summary, the experimental results demonstrate that the proposed model has significant advantages in feature extraction when dealing with lesions that have blurred and irregular edges. The model excels in handling complex lesion boundaries and blurred regions, showing stronger competitiveness. In the future, by focusing on lightweight model design, the efficiency and practical potential of the model could be further enhanced. Although our ablation experiments are conducted on skin lesion segmentation, the complementary effects of GGCAM and MHLFF suggest broader applicability. Specifically, GGCAM enhances global context modeling to address blurred lesion boundaries, while MHLFF focuses on multi-level feature fusion to preserve fine structural details. Such challenges are not unique to dermatology but also appear in other medical imaging tasks, such as brain tumor segmentation in MRI, lung nodule detection in CT, and breast lesion analysis in mammography. Therefore, the synergistic design of GGCAM and MHLFF has the potential to be generalized to a wider range of medical image segmentation problems that require both accurate boundary delineation and robust small-lesion detection. In future work, we plan to further explore this generalization by validating the modules on cross-domain datasets from multiple imaging modalities, which may provide deeper insights into their transferability and robustness in diverse clinical applications.

### 4.7 Robustness to hair occlusion: DMFF-Net vs. TransUNet

In order to further evaluate the robustness of DMFF-Net in challenging scenarios, we compared it with TransUNet on dermoscopic images containing severe artifacts such as hair occlusion and irregular illumination (Table 6). Quantitative results demonstrate that DMFF-Net achieves higher mIoU (91.47% vs. 90.51%), F-score (94.91% vs. 94.88%), recall (95.17% vs. 94.69%), and precision (95.76% vs. 95.51%), indicating superior segmentation reliability under artifact interference. Although TransUNet has fewer FLOPs (25.35G vs. 29.47G), its parameter count (105.28M) is significantly higher than that of DMFF-Net (4.25M), making our model more lightweight and efficient.

**Table 6:** Performance comparison between DMFF-Net and TransUNet on the ISIC 2017 dataset.

**No**.	**Model**	**MIoU**	**ACC**	**F1**	**Rec**.	**Prec**.	**Params**	**FLOPs**
1	TransUNet	90.51	98.65	94.88	94.69	95.51	105.28	25.35
2	Ours	91.47	97.33	94.91	95.17	95.76	4.250	29.47

Red-colored values indicate the best performance.

Visual comparisons (Figure 9b) further confirm this observation: while TransUNet often struggles to delineate lesion boundaries when hair artifacts are present, DMFF-Net preserves clearer and more accurate lesion contours, demonstrating stronger robustness to occlusion and lighting variation.

## 5 Discussion and limitations

In the work, a multi-scale and multi-attention feature fusion model, termed DMFF-Net, was developed based on DeepLabV3 to overcome the complex obstacles in skin lesion segmentation tasks. Through the integration of several innovative designs, DMFF-Net achieved outstanding performance across multiple publicly available skin lesion datasets, exhibiting significant advantages over existing mainstream networks.

Firstly, the experimental results clearly indicated that DMFF-Net exhibited notable robustness when handling skin lesion areas with complex shapes and irregular sizes. Unlike traditional single-scale convolutional networks, DMFF-Net effectively expanded the receptive field by introducing a MDSDC, allowing it to capture a broader range of contextual information. This was especially evident in cases where lesion boundaries were blurry or had low contrast. Traditional networks rely on fixed receptive fields, which complicates the task of balancing detailed information with global structure, leading to suboptimal segmentation performance. In contrast, our network combined multi-scale convolution with attention mechanisms, significantly improving segmentation accuracy. Secondly, GGCAM in DMFF-Net enhanced the network's ability to understand spatial dimension information by generating attention maps. Compared to existing networks, the GGCAM module more accurately captured and emphasized critical global features, avoiding the redundancy or loss of key information common in traditional methods. This design helped DMFF-Net better handle complex background interference in skin lesion segmentation, improving the model's balance between local detail and global semantic information.

Despite DMFF-Net's outstanding performance on multiple skin lesion datasets, there are still some limitations that require further improvement. Firstly, the complex network structure increases computational complexity and the number of parameters. Compared to classic models like U-Net and DeepLabV3, our network, due to the inclusion of multi-scale feature extraction and attention modules, may experience reduced performance under limited computational resources. In future research, our goal is to decrease the total parameters by adopting model compression and lightweight design techniques to improve the network's applicability. Additionally, although DMFF-Net has made significant improvements in handling blurry edges and irregularly shaped skin lesions, some segmentation errors still remain. As noted by Wang et al. (57), blurry edges and irregular shapes are common challenges in image segmentation. Future work could incorporate stronger edge detection mechanisms to further improve segmentation accuracy in these areas. Another limitation worth noting is the lack of diversity in current datasets representing real-world conditions. Although we achieved good results on the ISIC series datasets, these datasets do not fully represent all types of skin lesions encountered in clinical practice. Therefore, future studies will need to incorporate more diverse large-scale datasets, combined with transfer learning and self-supervised learning techniques, to further confirm and improve the model's resilience in practical scenarios.

## 6 Conclusion

In the work, we present an innovative network for feature fusion that employs multi-scale and multi-attention mechanisms, DMFF-Net, which is proposed, targeting the precise identification of skin lesion boundaries. The introduction of MHLFF and GGCAM modules enables the network to effectively integrate global and local feature information in the decoder stage, significantly enhancing its ability to segment complex skin lesions. Moreover, the MDSDC module improves the network's capacity to capture contextual information across multiple scales while preserving feature integrity at different levels. By combining global average pooling with channel and spatial attention mechanisms, DMFF-Net achieves a balanced integration of global and local features, further improving segmentation accuracy and robustness. Comprehensive experimental results on four public datasets, ISIC 2016, ISIC 2017, ISIC 2018, and *PH*^2^, demonstrate that DMFF-Net outperforms existing methods in the task of skin lesion segmentation, proving its ability to address challenges like indistinct lesion boundaries and irregular shapes. Through comparative experiments with other methods, we further verified the advantage of DMFF-Net in terms of accuracy. In the coming years, we intend to further decrease the number of parameters in the network while preserving segmentation accuracy. Our goal is to improve the model's capability to identify data with significant edge blurring and irregular lesion shapes, thereby enhancing both the accuracy and performance of the segmentation process. In addition, considering the practical clinical need for deployment on portable and resource-constrained devices, we plan to explore lightweight optimization strategies such as model quantization and pruning. These efforts will enable DMFF-Net to achieve efficient real-time inference on edge devices, thereby extending its potential application from research settings to real-world clinical environments.

## Data Availability

The original contributions presented in the study are included in the article/supplementary material, further inquiries can be directed to the corresponding authors.
